# High-Resolution Contact Localization and Three-Axis Force Estimation with a Sparse Strain-Node Tactile Interface Device

**DOI:** 10.3390/s26041378

**Published:** 2026-02-22

**Authors:** Yanyan Wu, Hanhan Wu, Yifei Han, Yi Ding, Bosheng Cao, Chongkun Xia

**Affiliations:** School of Advanced Manufacturing, Sun Yat-sen University, Shenzhen 518005, China; wuyy363@mail2.sysu.edu.cn (Y.W.); wuhh27@mail2.sysu.edu.cn (H.W.); hanyf33@mail2.sysu.edu.cn (Y.H.); dingy96@mail2.sysu.edu.cn (Y.D.); caobsh5@mail2.sysu.edu.cn (B.C.)

**Keywords:** sparse tactile sensing, contact localization, three-axis force estimation, sim-to-real transfer, few-shot fine-tuning

## Abstract

High-resolution contact localization and three-axis force estimation are crucial for human–robot interaction and precision manipulation, yet the sensing area is limited by channel density and wiring cost. Sparse strain readout makes joint estimation of location and three-axis force challenging due to cross-axis coupling and nonlinear responses, while dense arrays or extensive calibration increase complexity. We present a sparse strain-node tactile interface device (SSTID) whose three-module layout is optimized via particle swarm optimization to maximize informative response overlap, enabling contact localization (x,y) and three-axis force $\left(F_{x},F_{y},F_{z}\right)$ estimation using only nine strain channels. We further propose a strain-node contact-state decoding framework (SCDF) implemented with a lightweight multilayer perceptron and trained via a two-stage sim-to-real strategy, including FEM pretraining followed by few-shot real-data adaptation. Experiments demonstrate accurate contact-state decoding with full-workspace characterization, supporting low-cost and scalable deployment of sparse tactile interfaces.

## 1. Introduction

High-resolution contact localization and three-axis force estimation are indispensable for precise contact measurement and surface interaction [1,2,3]. Ideally, a tactile sensing system should not only measure the three-axis contact force vector but also localize the contact point accurately in space to support tasks such as contact-state estimation, force control, and slip feature analysis [4,5,6,7]. Conventional high-resolution tactile sensors often rely on dense arrays, which increase channel count, wiring complexity, packaging volume, and cost, and reduce reliability and cumbersome calibration procedures [8,9,10]. For deployable tactile systems, achieving perception capabilities comparable to high-resolution arrays under a “sparse sensing unit” constraint has become an important research direction in tactile sensing [11,12,13].

To alleviate the interconnect and packaging burden of high-density arrays, several alternative routes have been explored. Vision-based tactile sensors [14,15] can achieve high spatial resolution by tracking deformations of a compliant medium; however, they require imaging optics and additional packaging volume, which limits form-factor flexibility and robustness in compact deployments [16]. Moreover, learning a reliable mapping from observed deformations to force/location often demands large datasets or high-fidelity modeling, and performance is often sensitive to the domain gap between simulation and physical hardware [17]. To address this, Sferrazza et al. [18] proposed a sim-to-real strategy that generates training data in simulation and transfers it to real high-resolution optical tactile sensing, showing that “simulation-synthesized data and learning-based inference” can substantially reduce the burden of real data collection, while domain discrepancy remains a key factor affecting final accuracy and robustness.

A scalable alternative is computational tactile sensing, where mechanical transmission yields overlapping receptive fields and learning-based models reconstruct higher-resolution contact states from sparse readout. Recent super-resolution frameworks demonstrate that sparse tactile readout can still support dense virtual outputs when sensor layout and decoding models are co-designed [19]. Complementary theoretical [20,21] analyses further clarify the conditions and trade-offs for super-resolution under multidirectional loading, highlighting that stable multi-axis force and location decoding require careful handling of coupling, nonlinearity, and domain shift. Collectively, these studies suggest that sparse sensing units do not necessarily imply low spatial resolution if the structure and mapping model are properly designed; nevertheless, the stable decoding of the multidirectional force and contact location still require coupling, nonlinearity, and sim-to-real domain discrepancy to be addressed.

Among sparse tactile sensing modalities, strain-based sensing offers advantages such as compact structure, easy integration, fast response, and low cost, making it suitable for sparse deployment in confined spaces [22,23]. However, few-channel strain signals often exhibit pronounced cross-axis coupling and nonlinear responses, and the mapping between the contact location and three-axis force can drift with variations in material properties, assembly tolerances, bonding processes, and circuit gain. Recent studies on modular tactile units based on three-dimensional micro strain gauges have demonstrated the potential of micro-structured strain devices for multi-axis stimulus measurement and integration, while also highlighting the importance of multi-axis decoupling and consistent manufacturing for system performance [24]. On the other hand, low-cost tactile fingertips for robot end-effectors achieve contact localization and multi-DoF force/torque sensing via structural compliance and model-based inference, emphasizing deployability, integrability, and calibratability; yet such approaches still need to handle structural errors and calibration complexity [25,26]. Although finite element simulation can generate large-scale training data covering diverse conditions at relatively low cost, directly transferring the learned mapping from simulation to hardware is often limited by sim-to-real domain shift [27]. Conversely, relying entirely on large amounts of real data for end-to-end training or pointwise calibration significantly increases experimental cost, undermining the advantages of sparse and minimalist designs [28].

While prior sparse/super-resolution tactile studies commonly aim to reconstruct a dense virtual tactile map from sparse measurements (i.e., a super-resolution inversion problem) [19,20,21], our goal is different: we target contact-state inference under sparse strain readout by directly estimating the physically meaningful state. This leads to three key distinctions. (i) Decoding principle: instead of “sparse-to-dense” reconstruction that may amplify coupling and ambiguity under multi-axis loading, we formulate force–location decoupling as a unified regression-based contact-state inference problem, which directly outputs location and three-axis force without requiring an intermediate dense field. (ii) Training strategy: rather than depending on large-scale real calibration or purely simulation-trained models that degrade under domain shift, we adopt a two-stage sim-to-real pipeline that learns a physics-consistent prior from FEM data and then performs prior-constrained few-shot adaptation using a compact real dataset, explicitly mitigating systematic discrepancies. (iii) Hardware layout optimization: instead of using heuristic sensor placement or fixed layouts, we optimize the minimal three-module architecture and placement via PSO to maximize informative response overlap (i.e., identifiability) across the workspace under multi-directional loading, enabling channel-efficient decoding with only nine strain channels.

To address the strong coupling, nonlinearity, and sim-to-real domain shift that hinder high-resolution contact localization and three-axis force estimation under sparse strain readout, we jointly co-design the hardware structure and the decoding algorithm. Firstly, we design a compact sparse strain-node tactile interface device (SSTID) that adopts a three-module layout optimized via particle swarm optimization (PSO) to maximize effective response overlap across the workspace, enabling contact localization and three-axis force estimation using only nine strain channels. Building on this optimized structure, we first propose a strain-node contact-state decoding framework (SCDF) that formulates contact-state inference as a unified regression problem and is implemented with a lightweight multilayer perceptron (MLP). The framework adopts a two-stage sim-to-real strategy. Stage I pretrains the decoder on 14,400 FEM-simulated single-point contacts to learn a physics-consistent prior mapping of diverse contact locations, directions, and magnitudes. Stage II performs prior-constrained, regularized backpropagation using only 648 real calibration samples to compensate for systematic discrepancies caused by material properties, assembly tolerances, and circuit gain variations, thereby reducing the sim-to-real gap. With this pipeline, the proposed framework supports high-resolution contact-state decoding under sparse readout channels while maintaining low calibration cost and real-time deployability. More broadly, it offers a practical and scalable route toward low-cost, high-resolution three-axis tactile perception for contact-rich robotic sensing and control. The main contributions of this work are as follows:

(1) We establish a minimum three-module architecture and optimize its placement to maximize effective response overlap across the workspace, resulting in a channel-efficient SSTID with only nine strain channels over a 60 mm diameter workspace at a hardware cost below USD 15.
(2) We first propose an SCDF for force–location decoupling that employs a two-stage MLP training strategy. Stage I pretrains the decoder on 14,400 FEM-simulated contacts to learn a physics-consistent prior, and Stage II performs regularized few-shot fine-tuning with 648 real samples to reduce sim-to-real domain shift.
(3) We characterize its full-workspace accuracy and resolution, low force stability, repeatability/hysteresis/drift, and dynamic response, and further demonstrate sparse high-resolution three-axis force sensing via force regulation experiments.

## 2. Materials and Methods

### 2.1. Structural Optimization Design and Principal Analysis of the SSTID

To obtain the optimal channel design for the SSTID, we performed structural optimization. Under minimalist, low-cost hardware constraints, a key question is the minimum number of sensing modules required and how to place them to achieve stable and reproducible decoding of both the contact location and three-axis force. With fewer than three modules, the planar geometric reference frame becomes degenerate, making contact localization prone to multiple solutions or instability under noise. Therefore, we adopt a three-module configuration as the minimum setup that satisfies geometric non-degeneracy and provides sufficient information.

**Figure 1:**
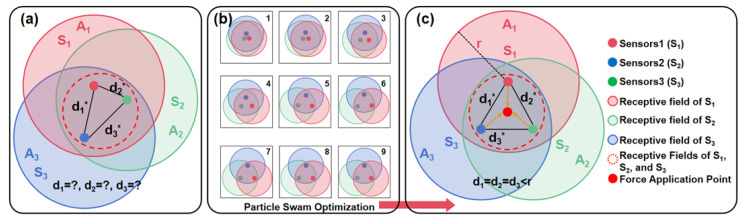
Optimization of the sensing units’ layout. (**a**) Problem statement for the optimization process. (**b**) Optimization process using the particle swarm optimization algorithm. (**c**) The optimization result, demonstrating that the optimal layout is achieved when $d_{1}=d_{2}=d_{3}=r$.

Under the three-module configuration, the joint identifiability of the contact location and three-axis force depends on two conditions: (i) the three modules must form a non-collinear geometric reference frame in the plane to avoid degeneracy in localization; and (ii) any contact within the workspace should be able to simultaneously excite at least three modules to produce effective responses—otherwise, the estimation becomes ill-conditioned due to insufficient information or noise amplification. Accordingly, as shown in Figure 1a, we define the “effective response region” of each module in the contact plane as a set. When the contact lies within $A_{i}$, the reaction force magnitude $F_{i}$ of module i exceeds a preset threshold and can be used for inference; outside this region, the response decays below the threshold and contributes negligibly to estimating location and load. The region $A_{i}$ can be approximated by a circular area with radius r based on a threshold isoline, where r is determined jointly by structural transmission characteristics and the chosen threshold and can be calibrated via experiments or simulations.

Based on the above definition, we assume that the three module centers form a triangle in the plane with pairwise distances $d_{1},d_{2},d_{3}$. To ensure that any workspace point can trigger effective responses from at least three modules, we enforce an overlap constraint that limits inter-module distances to be no larger than the effective radius r, and we maximize the union area of the three circular regions as a layout efficiency metric.

(1) $$\left(d_{1}^{*},d_{2}^{*},d_{3}^{*}\right)=\arg\;\max_{\left\{d_{1},d_{2},d_{3}\right\}}(A_{1}\cup A_{2}\cup A_{3}),\;subject\;to\;d_{1}\leq r,\;d_{2}\leq r,\;d_{3}\leq r$$

As shown in Figure 1b, we solve this optimization using particle swarm optimization (PSO) to obtain the optimal geometry under a given r. Under the general condition $r_{1}:r_{2}:r_{3}=1:1:1$, common optimizers converge to the same optimum: the three units lie at the vertices of an equilateral triangle with optimal side length r, yielding $d_{1}^{*}=d_{2}^{*}=d_{3}^{*}=r$, as shown in Figure 1c. This result directly supports the threefold-symmetric (0°, 120°, and 240°) module layout of the SSTID; while keeping the structure minimal, it maximizes the receptive field union and ensures simultaneous availability of informative signals across the workspace, thereby improving identifiability and reducing layout sensitivity for subsequent static decoupling and learning-based regression. This analysis provides a quantitative and reproducible basis for selecting a low-cost three-module layout.

**Figure 2:**
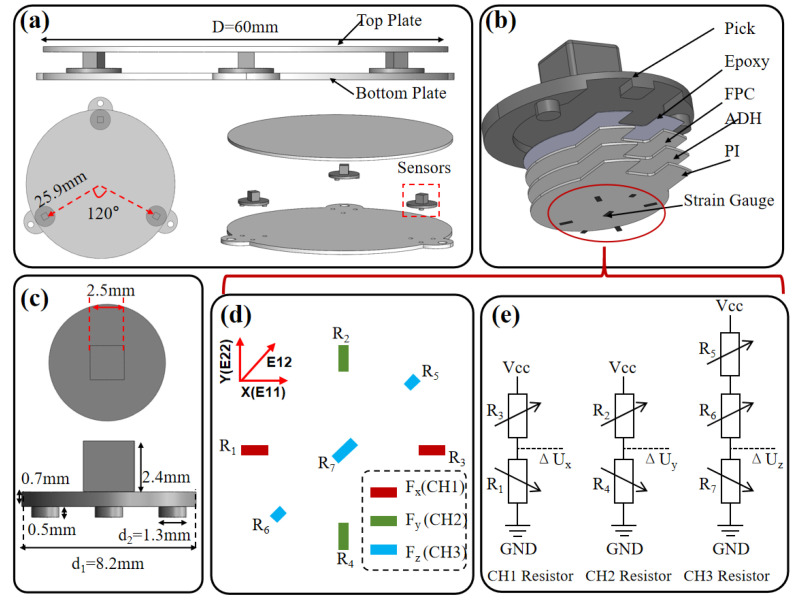
A schematic illustration of the structure and working principle of an SSTID. (**a**) A schematic illustration of the structure. (**b**) Geometric parameters of pick interface. (**c**) Strain gauge mounting architecture. (**d**) Strain gauge layout. (**e**) Wheatstone bridge circuit schematic.

Based on the layout analysis, the device adopts a threefold-symmetric configuration: the sensing plane consists of an SUS304 stainless steel top plate (diameter: 60 mm), three three-axis force sensing modules distributed circumferentially with 120° symmetry, and a rigid bottom plate made of the same material (Figure 2a). Both the top and bottom plates are 1 mm thick, and the top plate–sensor modules–bottom plate are bonded with metal adhesive to form an integrated force transmission chain (Figure 2b). Each sensing module uses a layered structure: a polycarbonate pick layer on top for load bearing (Pick), followed by a flexible printed circuit (FPC), acrylic pressure-sensitive adhesive (ADH), and a polyimide (PI) substrate. Seven metal foil strain gauges are integrated on the underside of the PI film (overall dimensions shown in Figure 2c). The strain gauges are arranged with functional zoning to enhance the separability of the three force components (Figure 2d): tangential gauges enhance the $F_{x}$ shear response, radial gauges enhance the $F_{y}$ shear response, and the central region enhances the $F_{z}$ normal response. When an external load is applied to the pick layer, deformation is transmitted through the FPC–ADH–PI stack to the strain gauge array, inducing resistance changes and forming strain observations correlated with three-axis forces, thereby providing a clear physical basis for cooperative decoding of the three-axis force and contact location. The total bill-of-materials cost is below USD 15, as summarized in Table 1. Importantly, this layout optimization not only maximizes channel efficiency but also improves the conditioning of the subsequent force–location decoupling model, thereby reducing sensitivity to noise and facilitating robust two-stage sim-to-real training.

### 2.2. Circuit Architecture and Readout Principle of the SSTID

The mechanical readout of the SSTID is based on the Wheatstone bridge principle, which converts strain gauge resistance variations into voltage signals for three-axis force prediction. As shown in Figure 2d,e, three orthogonally arranged strain gauge groups are integrated on the PI film, and each group adopts a half-bridge configuration to save space, corresponding to the measurement of the three force axes. When an external load is applied to the pick layer, mechanical deformation is transmitted through the FPC–ADH–PI laminate, inducing resistance changes $\Delta R_{i}$. The X- and Y-axis gauges each form a half-bridge, and the Z-axis gauges form a half-bridge with the corresponding resistors, as illustrated in Figure 2e. Here, $R_{1},R_{2},R_{3},R_{4},R_{7}$ share the same initial resistance R, while $R_{5}$ and $R_{6}$ are R/2. $\Delta R_{i}$ denotes the resistance change under deformation, and $\Delta R_{i}/R$ is the normalized resistance variation. The X- and Y-axis half-bridge output voltages $V_{\mathrm{out}\_x}$ and $V_{\mathrm{out}\_y}$ satisfy:

(2) $$V_{\mathrm{out}\_x}=V_{\mathrm{cc}}\left(\frac{1+\Delta R_{1}/R}{2+\Delta R_{1}/R+\Delta R_{3}/R}\right),\;\;V_{\mathrm{out}\_y}=V_{\mathrm{cc}}\left(\frac{1+\Delta R_{4}/R}{2+\Delta R_{4}/R+\Delta R_{2}/R}\right),$$

The Z-axis output voltage $V_{\mathrm{out}\_z}$ can be expressed as:

(3) $$V_{\mathrm{out}\_z}=V_{\mathrm{cc}}\left(\frac{1+\Delta R_{7}/R}{2+\Delta R_{7}/R+(\Delta R_{5}+\Delta R_{6})/R}\right),$$

The normalized variations $\Delta R_{i}/R$ for the X/Y/Z axes are jointly contributed by the averaged orthogonal strain components $E_{11}$ (length direction), $E_{22}$ (width direction), and $E_{12}$ (bisector direction), as shown in Figure 2d. Thus, $\Delta R_{i}/R$ can be written as:

(4) $$\frac{\Delta R_{i}}{R}=L\times E_{11}(R_{i})+W\times E_{22}(R_{i}),$$

where L and W are the sensor dimensions along the length and width directions, taking values 9.8 and 5.4, respectively. Finally, according to the strain gauge arrangement in Figure 2e, the three-axis signals corresponding to $F_{x}$, $F_{y}$, $F_{z}$ can be expressed as:

(5) $$Signal\_F_{x}=\delta \times (\frac{\Delta R_{1}}{R}-\frac{\Delta R_{2}}{R}),\;Signal\_F_{y}=\delta \times (\frac{\Delta R_{3}}{R}-\frac{\Delta R_{4}}{R}),\;Signal\_F_{z}=\delta \times (\frac{\Delta R_{5}+\Delta R_{6}}{R}-\frac{\Delta R_{7}}{R})$$

Here, δ is the bridge excitation voltage and is set to 2.8 V, enabling conversion of the applied load on a single sensor module into voltage signals.

### 2.3. Design of the Simulation Dataset

**Figure 3:**
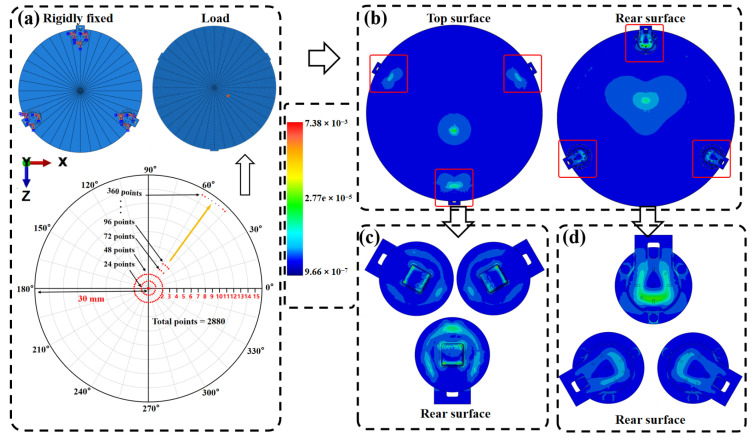
FEM simulation setup and dataset generation for the SSTID. (**a**) Prescribed sampling of single-point contact locations and three-axis forces over the workspace, with magnitudes spanning 0–5 N and directions covering normal and tangential loading. (**b**) Representative FEM-predicted response under applied loading with boundary constraints matching the experimental fixture. (**c**) Top-side loading cases. (**d**) Bottom-side loading cases. The responses at the nine strain readout nodes are extracted to form the Stage I pretraining dataset.

**Figure 4:**
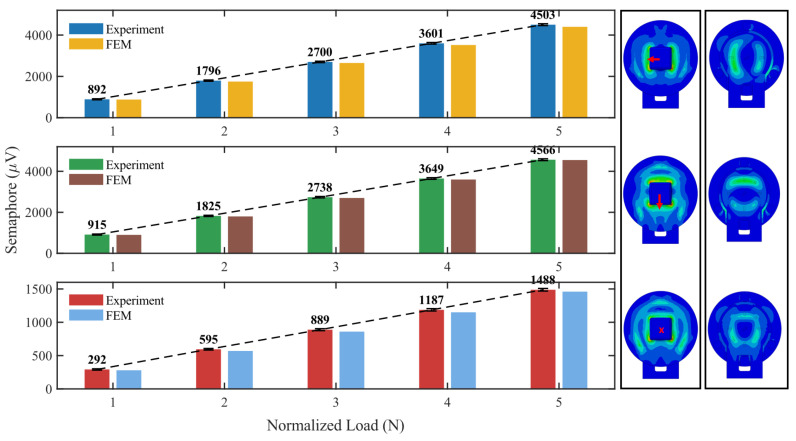
A comparison of the accuracy of the SSTID’s $\left(F_{x},F_{y},F_{z}\right)$ simulation and experimental results in the 0–5 N range.

To enable Stage I pretraining of the force–location decoder and to provide a physics-consistent prior that is transferable to real hardware, we construct a large-scale FEM simulation dataset covering diverse contact locations, directions, and magnitudes. We perform batch FEM simulations by applying concentrated loads with varying directions and magnitudes on the surface of the tactile interaction platform and obtain the corresponding three-axis force responses of the three sensors. Following the procedure in Figure 3a, we construct a dataset of 14,400 samples: 7200 samples with random three-axis force loading in the range 0–5 N, and another 7200 samples with random normal-force loading in the range 0–5 N, both applied at random locations on the platform surface. Figure 3b–d show the stress and strain distributions of the top plate and individual modules under representative loading cases, supporting the consistency of the load–transmission–strain response chain in the FEM model. Loads in different directions induce distinctive response patterns across the three modules, providing discriminative information for subsequent regression of both (x,y) and $\left(F_{x},F_{y},F_{z}\right)$ from few-channel signals. We validate fidelity at both the module and system levels. Figure 4 shows a quantitative FEM experiment comparison of the module-level output voltage differences, where the $F_{z}$-related (normal) channel and the $F_{x}/F_{y}$ related (tangential) channels match closely under the same loads. For the assembled three-module SSTID, we further apply 1 N normal loads at 73 spatial points and obtain an average relative error of 11% for $F_{z}$, while the average tangential-force error ($F_{x}/F_{y}$) is 32%, mainly due to assembly tolerances and inter-module coupling not fully captured by FEM.

To ensure physically consistent and transferable simulation data, we used the elastic moduli in Table 2 for the pick, epoxy, ADH, FPC, PI, and strain gauges, while keeping geometry and boundary conditions consistent with the physical assembly. This configuration was used to generate pretraining data and to provide a physics-consistent prior for the SSTID.

### 2.4. Real-World Data Collection Protocol

**Figure 5:**
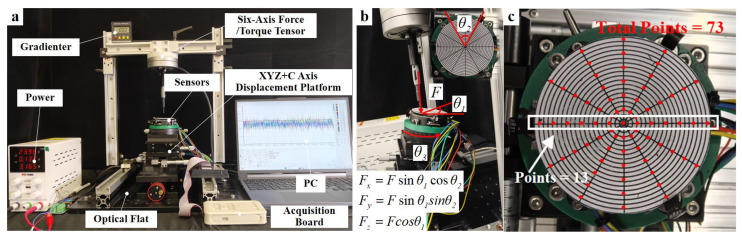
SSTID experimental setup. (**a**) Calibration platform; (**b**) inclined force application platform; (**c**) distribution of data collection points on the top surface of the SSTID.

To acquire real data and validate the performance of the SSTID, we designed and fabricated a measurement setup as shown in Figure 5a. The setup consists of a three-DoF gantry stage and a high-precision base with five degrees of freedom (X, Y, Z, rotation, and tilt), controlled via adjustment knobs. The knob resolution for X/Y/Z translation is 0.02 mm per division, and the tilt knob resolution is 0.1° per division. A force/torque (F/T) sensor (NANO25, ATI Industrial Automation) was used as the reference, with rated loads of $F_{x},F_{y},F_{z}$: 100 N and $M_{x},M_{y},M_{z}$: 4 N·m. The F/T sensor data, together with all other measurement data, were transmitted to a computer for recording.

For low-cost sim-to-real adaptation, we collected N=648 real samples for backpropagation-based adjustment. We selected 73 spatial points on the sensing plane (computed as 13 × 6 − 5 = 73), where the center point is counted only once across the six radial groups. Each point included two loading types: normal loading and inclined loading.

(1) Normal loading (216 samples): At each spatial point, a normal force $F_{z}$ ∈ {1,3,5}  N was applied, resulting in 216 normal force samples in total.

(2) Inclined loading (432 samples): At each spatial point, six inclined force samples were applied, resulting in 432 samples in total. The inclined force vector was parameterized by magnitude–tilt angle–azimuth angle: the magnitude $F_{total}$ ∈ [0,5]  N; the tilt angle θ is the angle between the force vector and the normal Z axis; and the azimuth angle ϕ is the in-plane angle of the force vector relative to the X axis. The components are:

(6) $$F_{x}=F\sin\theta \cos\phi ,\;\;\;\;F_{y}=F\sin\theta \sin\phi ,\;\;\;\;F_{z}=F\cos\theta$$

Accordingly, the normal-force component is denoted as $F_{z}$ (and the force vector as $F=[F_{x},F_{y},F_{z}]$) throughout this work. Given the constraint of only six inclined loadings per point, we adopted a uniform coverage strategy with two tilt angles and three azimuth angles to improve generalization across shear directions and shear-to-normal ratios: ϕ∈ {0°,120°,240°}, and θ ∈ {30°,60°}. Meanwhile, to avoid spurious correlations caused by fixed binding between the direction and magnitude, the magnitudes {1,3,5}  N were cyclically permuted with the azimuth angles across different spatial points (a cyclic right shift of {1,3,5} according to point index), achieving balanced coverage of direction, shear ratio, and magnitude over the full plane.

The real calibration set (648 samples) is designed to be representative rather than exhaustive. This sampling strategy covers combinatorial variations in shear direction, shear-to-normal ratio, and magnitude with minimal trials per point: three azimuth angles cover the principal in-plane shear directions, and two tilt angles cover different shear proportions. The cyclic permutation between magnitudes and azimuth angles further reduces pseudo-correlation due to fixed magnitude–direction pairing, improving model generalization under a limited total sample budget and enhancing the efficiency of real-world adjustment.

### 2.5. Learning-Based SCDF with Two-Stage Sim-to-Real Adaptation

Based on the simulation and real calibration datasets introduced above, we next present the proposed strain-node contact-state decoding framework (SCDF) for mapping nine-channel strain observations to a continuous contact location and three-axis force. To bridge the sim-to-real gap while keeping calibration cost low, the SCDF is trained using a two-stage strategy: simulation pretraining for a physics-consistent prior followed by regularized few-shot adaptation on real data. We first define the input as a 9-dimensional displacement/strain feature vector $s\in {\mathbb{R}}^{9}$ and the output as a 5-dimensional target vector:

(7) $$y={\left[x,y,F_{x},F_{y},F_{z}\right]}^{T}\in {\mathbb{R}}^{5},$$

where (x,y) denotes the contact location and $\left(F_{x},F_{y},F_{z}\right)$ denotes the three-axis force components. The regression model is $\hat{y}=f(s;\theta )$. To mitigate the influence of scale differences across dimensions during optimization, both inputs and outputs are standardized using a Standard Scaler:

(8) $$\bar{s}=(s-\mu_{s})/\sigma_{s},\;\;\;\;\bar{y}=(y-\mu_{y})/\sigma_{y},$$

the network regresses $\bar{y}$ in the standardized space, and the outputs are de-standardized during inference to obtain physical quantities.

As illustrated in Figure 5, the SCDF is implemented as a lightweight multilayer perceptron (MLP) decoder composed of stacked fully connected blocks with nonlinear activations and a final linear output layer. This architecture is sufficient to model the strongly coupled and nonlinear mapping from few-channel strain signals to multi-dimensional contact states while remaining computationally efficient for real-time deployment.

To address the sim-to-real domain shift caused by material properties, assembly tolerances, and circuit gain variations, the SCDF adopts a two-stage sim-to-real adaptation strategy. In Stage I (simulation pretraining), we train the decoder on the FEM-based simulation dataset D_sim (14,400 samples) to obtain $\theta_{0}$ which serves as a physics-consistent prior mapping. In Stage II (few-shot real adaptation), we initialize the model with $\theta_{0}$ and continue training on the real calibration dataset D_real (N = 648), using prior-constrained and regularized optimization to absorb systematic hardware discrepancies into the model parameters and reduce the sim-to-real gap. The Stage II parameter update can be written as:

(9) $$\theta \leftarrow \theta_{0}-\eta \nabla_{\theta }L_{ft},$$

thereby absorbing systematic errors caused by material, assembly, and circuit gain discrepancies into the model parameters and reducing the sim-to-real gap. The model output is grouped into location and force terms, and a weighted regression loss is adopted:

(10) $$L_{task}=\lambda_{p}{\left\|\tilde{p}-\bar{p}\right\|}_{2}^{2}+\lambda_{f}{\left\|\tilde{f}-\bar{f}\right\|}_{2}^{2},$$

where $p={\left[x,y\right]}^{⊤}$ and $p={\left[x,y\right]}^{⊤},\;f={\left[F_{x},F_{y},F_{z}\right]}^{⊤}$. With standardized outputs, we typically set $\lambda_{p}=\lambda_{f}=1$. To suppress overfitting during few-shot adjustment and explicitly preserve the simulation prior, we introduce an L2-SP regularization term during fine-tuning:

(11) $$L_{ft}=L_{task}+\beta {\left\|\theta -\theta_{0}\right\|}_{2}^{2},$$

where β is the regularization weight. We determine β via a log-scale sweep on a held-out validation split of the real calibration set (e.g., $\beta \in \left\{10^{-5},10^{-4},10^{-3},10^{-2},10^{-1}\right\}$) and select the value that minimizes validation error; a sensitivity check confirms stable performance within a reasonable range, while too small β overfits few-shot real data and too large β under-adapts to the real domain. In this work, we use $\beta =10^{-3}$ unless otherwise stated.

Both stages employ mini-batch optimization; pretraining uses a larger learning rate for fast convergence, whereas fine-tuning adopts a smaller learning rate and validation-based early stopping to improve generalization. During inference, the SCDF consists only of fully connected operations and element-wise activations, enabling real-time output of $\left(x,y,F_{x},F_{y},F_{z}\right)$ on conventional edge-computing platforms. Detailed training hyperparameters and implementation settings are provided in Section 2.6.

### 2.6. Training Procedure

**Figure 6:**
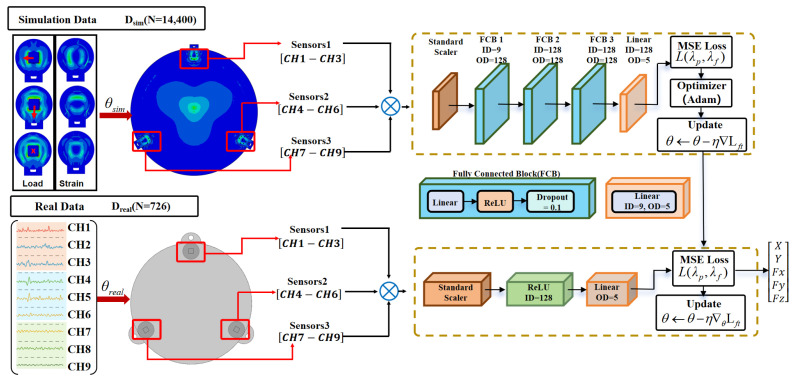
Model training SCDF diagram.

**Figure 7:**
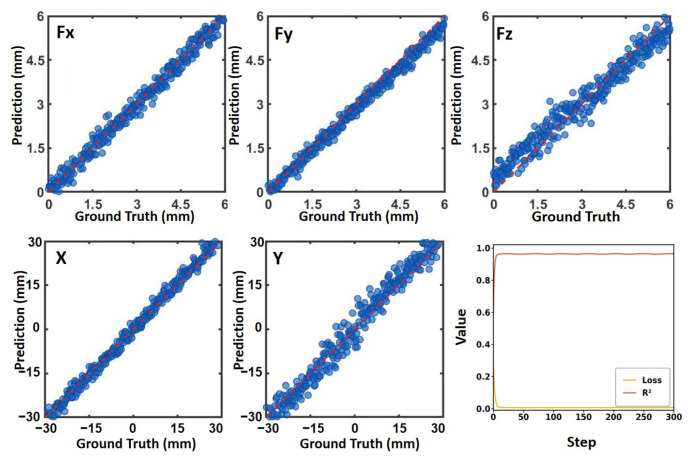
Training results and loss curve.

Based on the model definition and two-stage objectives in Section 2.5, we detail the implementation settings for Stage I simulation pretraining and Stage II few-shot real adaptation, including data splits, optimization settings, and training criteria. For Stage I, the FEM-simulated dataset of 14,400 single-point contacts was randomly split into 80% training, 10% validation, and 10% test. For Stage II, the real calibration dataset of 648 samples was split using the same 90%/10% protocol. Unless otherwise stated, the training pipeline and hyperparameters were identical for both stages. To balance loss contributions across dimensions and improve optimization stability, both inputs and outputs were standardized (z-score normalization). During training, the standardized inputs were forwarded through the MLP, and a sequence of fully connected linear mappings with ReLU activations produced predictions $\hat{y}$ for $\left[x,y,F_{x},F_{y},F_{z}\right]$. The mean squared error loss $L(\hat{y},y)$ was then computed against the ground truth labels. Backpropagation was performed from this loss, and PyTorch automatic differentiation computed gradients of the loss with respect to the weights and biases of each layer via the chain rule (with gradient gating through ReLU derivatives). For each mini-batch, gradients were cleared, recomputed, and parameters were updated using the Adam optimizer (learning rate 10^−3^), iteratively driving model outputs toward the ground truth. The model was implemented using PyTorch 2.6.0 under Python 3.12 and training converged within 300 epochs. As shown in Figure 6 and Figure 7, the model achieved a mean squared error (MSE) = 0.011, mean absolute error (MAE) = 0.092, and $R^{2}=0.981$ on the test set, indicating good prediction accuracy and generalization. The predicted-versus-ground-truth scatter plots for each component closely align with the diagonal, demonstrating strong overall consistency across multi-dimensional outputs. Meanwhile, the loss curve stabilizes in the later stage of training and the $R^{2}$ curve converges close to 1, suggesting no noticeable oscillation or degradation during optimization. These results provide a reliable baseline for subsequent real-world adaptation and metrological experiments.

## 3. Results

**Figure 8:**
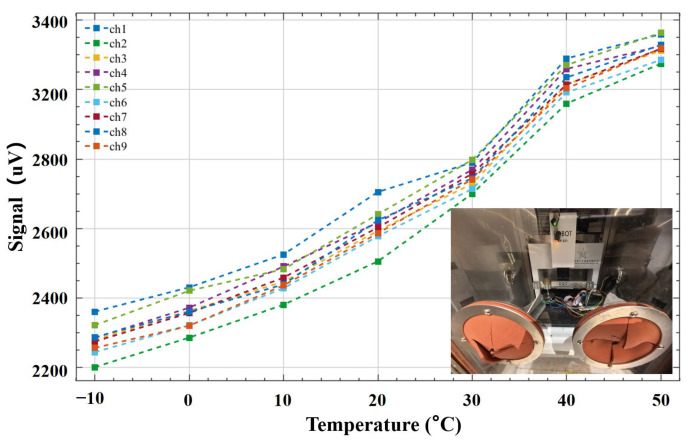
Variation curves of sensor signals from −10 °C to 50 °C.

**Figure 9:**
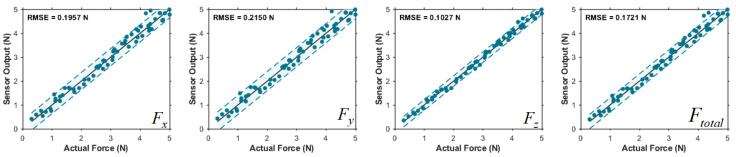
Mean error of the three-axis forces and the resultant force.

**Figure 10:**
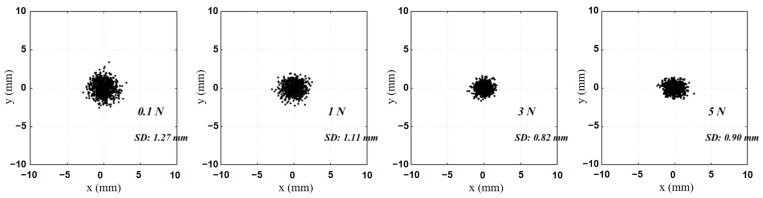
Distribution of measured contact points according to the applied contact force.

**Figure 11:**
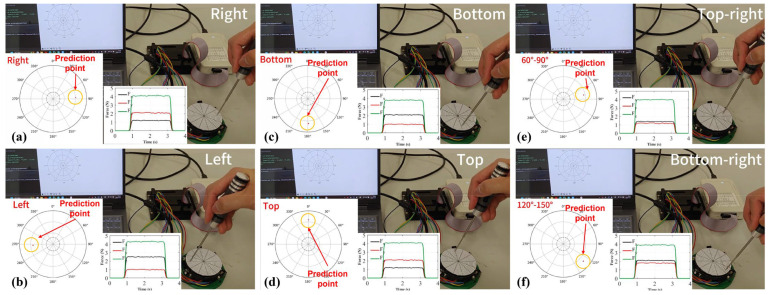
Contact localization experiment of the SSTID. (**a**) Right; (**b**) Left; (**c**) Bottom; (**d**) Top; (**e**) Top-right; (**f**) Bottom-right.

**Figure 12:**
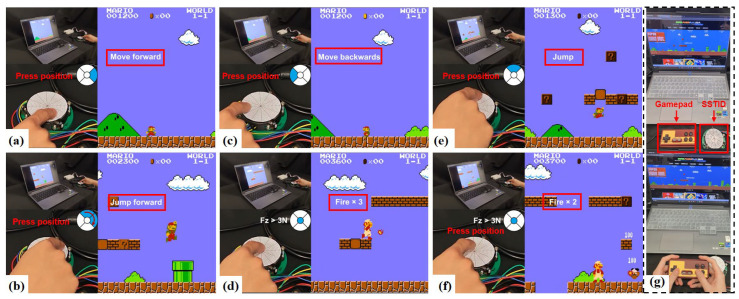
Playing Super Mario using the SSTID. (**a**) Move forward; (**b**) Jump forward; (**c**) Move backwards; (**d**) Release three attacks; (**e**) Jump; (**f**) Release two attacks; (**g**) Comparison diagram of playing games with controller and SSTID.

This section evaluates the sensor in terms of: force-estimation accuracy, stability of contact localization, key metrological metrics, and application demonstrations. We first report the evaluation of temperature drift (Figure 8) and then by the mean errors of the three-axis forces and the resultant force (Figure 9), then analyze the distribution stability of estimated contact points under different load levels (Figure 10) for the SSTID through a few-shot adaptation ablation study (Table 3) and demonstrate real-time usability in interaction and game-control scenarios (Figure 11 and Figure 12), and summarize core metrics such as full-scale range, sensitivity, resolution, repeatability, and hysteresis in Table 4. Finally, we evaluate the benefit of real calibration by varying the number of real samples used in Stage II fine-tuning and reporting the corresponding changes in contact localization and force estimation errors.

### 3.1. Performance Evaluation of the SSTID

Table 4 summarizes key metrics including full-scale output (FSO = 5 N), sensitivity, force resolution, repeatability, and hysteresis. Repeatability error was computed from the output dispersion under repeated cyclic loading at the same load level, while hysteresis was computed from the output difference between loading and unloading cycles at the same force level. These metrics evaluate the engineering usability of the SSTID from a metrological perspective, beyond pure regression accuracy. As shown in Table 4, repeating a 5 N load over 100 cycles produced consistent signal variations, and the computed average repeatability error was 1.3% of FSO, indicating stable dynamic behavior and strong mechanical robustness. To analyze hysteresis, we applied a constant 5 N load to the sensor for 10 loading/unloading trials; the results show an average hysteresis of approximately 3.1% (Table 4).

Table 3 summarizes the decoder ablation and rationale. Since the SCDF maps a compact strain vector (nine channels) to the continuous contact state, we benchmark the proposed MLP decoder against classical regressors (LR and KNN). All baselines are trained and tuned on the same real calibration split via cross-validation. We note that these classical models cannot directly leverage our Stage I physics-consistent pretraining and Stage II prior-constrained adaptation in a unified differentiable pipeline; when trained on FEM data, they suffer from sim-to-real discrepancy. Table 3 shows that the MLP provides the best accuracy–latency trade-off, supporting its selection for real-time deployment. We additionally evaluate performance versus the number of real samples (e.g., 162/324/486/648) and observe diminishing returns as the sample size increases, supporting the adequacy of 648 samples. Nevertheless, we note that insufficient real samples may underrepresent extreme loads or local regions, which we list as a limitation and will address in future work with broader real data coverage.

Figure 8 presents the temperature drift characteristics of the SSTID. Experiments were conducted in a programmable temperature–humidity chamber (ST-1000L, ZhenHang, Shenzhen, China) with a controllable temperature range of −70 °C to 150 °C and an accuracy of ±0.5 °C. The SSTID was placed in the chamber and the ambient temperature was swept from −10 °C to 50 °C in 10 °C increments, while the channel outputs were recorded. The nine-channel outputs exhibited an approximately linear dependence on temperature, with an average drift magnitude of about 300 μV per 10 °C. This drift propagated to the decoded outputs: for each 10 °C temperature change, the estimation accuracy of $F_{x},F_{y}$ and $F_{z}$ deteriorated by approximately 0.117 N, 0.102 N, and 0.191 N, respectively; meanwhile, the contact location error increased by about 0.13 mm per 10 °C. To mitigate the temperature-induced drift observed in Figure 8, we apply a software-based online drift suppression in signal preprocessing. Specifically, the raw strain sequence $x_{i}$ is smoothed using a sliding-window mean filter with window length W(W = 6 in this work): for the first samples we use a variable-length window $y_{i}=\frac{1}{i}\sum_{j=1}^{i}x_{j}$ when i<W, and afterward a fixed window $y_{i}=\frac{1}{W}\sum_{j=i-W+1}^{i}x_{j}$ when i≥W. This operation tracks and attenuates the slowly varying baseline component caused by thermal drift, improving output stability during long-duration operation. Note that this is a lightweight drift suppression rather than an explicit temperature model; in future work, we will incorporate on-board temperature sensing and temperature–strain co-calibration (or domain-adaptive learning) for more rigorous compensation.

Figure 9 reports the prediction errors (annotated by RMSE) of the three-axis forces $\left(F_{x},F_{y},F_{z}\right)$ and the resultant force $F_{total}$. The errors are of a similar magnitude across axes, and the resultant-force error does not exhibit noticeable amplification, indicating that the model maintains good vector consistency under multi-axis coupled loading. This suggests that the physics-consistent mapping learned via simulation-prior pretraining, together with few-shot real-world adjustment that compensates for systematic biases, effectively suppresses cross-axis error propagation induced by material and assembly variations. We further examined the distribution of estimated contact points under different contact-force levels. As shown in Figure 10, when a constant force of 0.5 N, 1 N, 3 N, and 5 N was applied at a fixed point on the cover plate for 10 s, the standard deviation (SD) of estimated contact points was 1.32 mm at 0.5 N. At 1 N and 3 N, the SD values were 1.06 mm and 0.82 mm, respectively. At 5 N, the estimated contact point remained stable with SD = 0.87 mm.

Figure 11 shows real-time localization and force estimation under different contact regions and loading directions. The correctness of location decoupling is validated by the relative relationship between the predicted point and the ground truth contact region, and the dynamic tracking ability under pressing and direction changes is verified by the temporal responses of the three force components. This experiment highlights that the sensor not only outputs a scalar force but also achieves simultaneous decoding of “contact location + force vector” under sparse-channel constraints. See Supplementary Movie S1 for related experiments.

### 3.2. Demonstration in Application Scenarios

Figure 12 demonstrates a human–machine interaction application based on real-time tactile decoding. By mapping the predicted contact location to directional control and mapping $F_{z}$ to a button-trigger threshold, the user can control character movement, jumping, and attacking in the game. This demonstration validates the stability and real-time capability of the decoded outputs from an application perspective and forms a closed loop with the “compatible control types” summarized in Table 5. See Supplementary Movie S2 for details.

In Figure 12, the decoded continuous contact state is mapped to discrete game commands by assigning the predicted contact location to directional inputs and assigning the decoded force component to button-trigger events, forming a closed-loop human-in-the-loop interface. To objectively quantify practicality beyond qualitative visualization, we report the quantitative metrics summarized in Table 6, including task-level performance (success rate and completion time under a fixed level segment), command-level accuracy (direction accuracy and button-trigger $F_{z}$, evaluated by time-aligned comparison between the SSTID-generated command stream and a reference stream from a standard gamepad or scripted commands, together with the mis-press rate in events/min), and latency (mean end-to-end latency measured from the decoded trigger threshold crossing $t_{0}$ to the OS-level key event timestamp $t_{1}$, as well as the in-game latency reported during gameplay). These results provide an objective and reproducible assessment of the game control interface beyond qualitative visualization.

A key advantage of the SSTID lies in its channel efficiency, i.e., how large a tactile workspace can be covered per electrical readout channel while still enabling multi-dimensional inference. As shown in Table 7, conventional strain gauge tactile units typically operate over millimeter-scale active areas, and multi-axis load sensing often requires multiple strain gauges per unit, leading to a small area-per-channel ratio. Here, the “channel efficiency” is used to describe the sensing coverage achieved per electrical readout channel, in order to reflect the coverage–wiring trade-off of sparse tactile interfaces. In this manuscript, it is computed as the effective workspace area divided by the number of independent strain channels. For our SSTID, the workspace is a circular region with a diameter of 60 mm (total area 28.3 cm^2^) and the readout uses nine channels, corresponding to 3.14 cm^2^ coverage per channel. The same definition is adopted for the entries summarized in Table 7 whenever the workspace size and channel count are available in the cited references. In contrast, our system uses only nine strain channels to achieve tactile perception over a circular workspace with a 60 mm diameter (28.3 cm^2^). This corresponds to 3.14 cm^2^ per channel, while simultaneously estimating the contact location (x,y) and three-axis force $\left(F_{x},F_{y},F_{z}\right)$.

## 4. Conclusions

High-resolution contact localization and three-axis force estimation are crucial for human–robot interaction and precision manipulation, yet this capability is fundamentally limited by channel density and wiring cost. With sparse strain readout, low-dimensional measurements make decoupling of the location and three-axis force susceptible to cross-axis coupling and nonlinear responses. We address high-resolution contact localization and three-axis force estimation under sparse strain readout by jointly co-designing the hardware structure and decoding the algorithm. Specifically, we perform a layout optimization using particle swarm optimization (PSO) to determine the minimum three-module configuration and its optimal placement, maximizing response overlap and yielding a channel-efficient SSTID with only nine strain channels. Building on this optimized structure, we propose an SCDF that casts force–location decoupling as a unified regression problem and is realized with a lightweight multilayer perceptron (MLP). The decoder is trained with a two-stage sim-to-real strategy, where Stage I learns a physics-consistent prior from 14,400 FEM-simulated single-point contacts and Stage II performs prior-constrained, regularized adaptation using 648 real calibration samples to compensate for systematic discrepancies from material properties, assembly tolerances, and circuit gain variations.

Systematic metrological characterization and ablation studies validate accuracy and resolution across the workspace and quantify low-force stability, repeatability, hysteresis, and dynamic response. Within a 5 N full-scale range, the force resolution reaches 5 mN, with an average repeatability error of 1.3% FSO and an average hysteresis of 3.1% FSO. With N=648 real samples for Stage II, the position MAE decreases to 1.97 mm and the three-axis force MAE decreases to 0.17 N, confirming the effectiveness of combining simulation-based prior learning with few-shot real adaptation. With a hardware cost below USD 15, the SSTID provides a practical reference for cost-sensitive deployment of three-axis force and contact localization in contact-rich robotic sensing and control. Looking forward, we will pursue cross-device generalization and establish an interpretable link between sensor scale and structural parameters and the resulting force–location response characteristics, enabling consistent calibration and rapid transfer across devices with different sizes and manufacturing variations.

## Figures and Tables

**Table 1:** The cost of the SSTID.

Part	Quantity	Unit Price (USD)	Total Price (USD)
Sensor Module	3	1.1	3.3
Microcontroller	1	5.2	5.2
Circuit Board	3	1	3
Top Plate	1	1	1
Bottom Plate	1	1	1
Base	1	0.5	0.5
Electrical Wire	1	1	1
Total Cost			15

**Table 2:** Material properties used in FEM simulation.

Part Name	Value (MPa)
Pick	19,000
Epoxy	250
ADH	600
FPC	4100
PI	5000
Strain gauges	90,000

**Table 3:** Ablation study.

Method	Position MAE (mm)	Three-Axis Force MAE (N)
MLP (only simulation)	10.2	0.51
$\mathrm{MLP}+\mathrm{Backpropagation}\;(N=216\;F_{z})$	3.51	0.35
$\mathrm{MLP}+\mathrm{Backpropagation}\;(N=216\;F_{z}+144\;F_{x}\;and\;F_{y})$	2.87	0.27
$\mathrm{MLP}+\mathrm{Backpropagation}\;(N=216\;F_{z}+288\;F_{x}\;and\;F_{y})$	2.38	0.21
MLP+Backpropagation (N=648)	1.97	0.17
KNN (K = 5, only sim data)	12.1	0.69
KNN (K = 5, only real data)	3.01	0.38
Linear Regression (only sim data) Linear Regression (only real data)	15.3 3.59	0.75 0.43

**Table 4:** Sensor performance characterization.

Category	Quantity	Value	Unit
Specifications	Force	5	N
	Sensitivity	102	N^−1^
	Force resolution	5	mN
Performance metrics	Error of average repeatability	1.3	% of FSO
	Average hysteresis	3.1	% of FSO
	$F_{z}$ axis 50,000 cycles of 1 N load test	19	% initial value error

Note: FSO denotes the full-scale output (5 N). “% initial value error” denotes the relative deviation from the initial reading measured after the 50,000-cycle test under a 1 N load.

**Table 5:** The sensor is compatible with game controls.

Game Genre	Controls	SSTID Adaptation
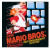 Super Mario	D-pad movement + button jump/attack	↑↓←→ + $F_{z}$
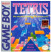 Tetris	D-pad rotation + quick drop	↑↓←→ + $F_{z}$
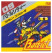 Tank Battle	Stick move + fire	↑↓←→ + $F_{z}$
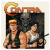 Shooter (Contra)	D-pad movement + button jump/attack	↑↓←→ + $F_{y}$ + $F_{z}$

**Table 6:** Quantitative evaluation of the game-control demonstration and press-trigger reliability.

Evaluation Block	Protocol	Trials	Metric	Placeholder Result
Mario task level (SSTID)	Fixed level segment	20	Success rate (%)	90
		20	Completion time (s), mean ± std	41.8 ± 6.5
Mario task level (Gamepad)	Same level segment; standard gamepad (two-hand)	20	Completion time (s), mean ± std	38.5 ± 5.4
Mario command level	Time-aligned command stream comparison (direction + button)	20	Direction accuracy (%)	95.6
Latency	$t_{0}$: decoded threshold crossing;	200	Mean latency (ms), mean ± std	60 ± 12
	$t_{1}$: OS key event timestamp	200	Games latency (ms)	91
Press-based trigger reliability	Short-press: single-trigger; long-press: hold 3 s	100	Short-press trigger success (%)	98
98		50	Long-press trigger success (%)	100

**Table 7:** Comparative study.

Work	Sensing Principle	Readout Channels	Active Area (cm^2^)	Active Area/Channel (cm^2^/ch)	Outputs	Reported Performance
This work	Sparse strain sensing + learning	9	28.3	3.14/channel	$\left(x,y,F_{x},F_{y},F_{z}\right)$	Position MAE 1.97 mm; three-axis force MAE 0.17;
Kong et al. [19]	Sparse taxels + deep model	23	47.52	2.066	High-res pressure map/localization	Average localization error 0.73 mm;
Piacenza et al. [28]	Conductive elastomer + multi-electrode	6	1.6	0.024 /line	Localization + depth	median localization accuracy; depth error ~0.74 mm;
Lee et al. [29]	Strain gauges on micro-structure	8	0.027	0.027	Three-axis force	Triaxial sensing (unit-scale)
Mêda et al. [30]	Array + MRE skin + neural network	16	6.5	0.41	$\left(x,y,F_{x},F_{y},F_{z}\right)$	Location MAE 0.26 mm Force magnitude MAE 0.17 N;

## Data Availability

The original contributions presented in this study are included in this article; further inquiries can be directed to the corresponding author.

## Supplementary Materials

The following supporting information can be downloaded at: https://www.mdpi.com/article/10.3390/s26041378/s1, Supplementary Materials, Video S1 and Video S2.
